# Association between Adherence with Recommended Antenatal Care in Low-Risk, Uncomplicated Pregnancy, and Maternal and Neonatal Adverse Outcomes: Evidence from Italy

**DOI:** 10.3390/ijerph18010173

**Published:** 2020-12-29

**Authors:** Giovanni Corrao, Anna Cantarutti, Anna Locatelli, Gloria Porcu, Luca Merlino, Simona Carbone, Flavia Carle, Rinaldo Zanini

**Affiliations:** 1 National Centre for Healthcare Research and Pharmacoepidemiology, Department of Statistics and Quantitative Methods, University of Milano-Bicocca, 20126 Milan, Italy; giovanni.corrao@unimib.it (G.C.); gloria.porcu@unimib.it (G.P.); f.carle@staff.univpm.it (F.C.); 2 Unit of Biostatistics, Epidemiology and Public Health, Department of Statistics and Quantitative Methods, University of Milano-Bicocca, 20126 Milan, Italy; 3 Department of Obstetrics and Gynecology, ASST Vimercate, Vittorio Emanuele III Hospital, University of Milano-Bicocca, Monza-Brianza, 20126 Milan, Italy; anna.locatelli@unimib.it; 4 Welfare Department, Epidemiologic Observatory, Lombardy Region, 20121 Milan, Italy; luca.merlino@cardiologicomonzino.it; 5 Department of Health Planning, Italian Health Ministry, 5–00144 Rome, Italy; s.carbone@sanita.it; 6 Center of Epidemiology and Biostatistics, Polytechnic University of Marche, 60121 Ancona, Italy; 7 Woman and Child Health Department, Azienda Ospedaliera della Provincia di Lecco, 23900 Lecco, Italy; rinaldozanini@gmail.com

**Keywords:** antenatal care, maternal outcomes, neonatal outcomes, periodic examinations, pregnancy, recommendations

## Abstract

Antenatal care (ANC) aims of monitoring wellbeing of mother and foetus during pregnancy. We validate a set of indicators aimed of measuring the quality of ANC of women on low-risk, uncomplicated pregnancy through their relationship with maternal and neonatal outcomes. We conducted a population-based cohort study including 122,563 deliveries that occurred between 2015 and 2017 in the Lombardy Region, Italy. Promptness and appropriateness of number and timing of gynaecological visits, ultrasounds and laboratory tests were evaluated. We assessed several maternal and neonatal outcomes. Log-binomial regression models were used to estimate prevalence ratio (PR), and corresponding 95% confidence interval (95% CI), for the exposure→outcome association. Compared with women who adhered with recommendations, those who were no adherent had a significant higher prevalence of maternal intensive care units admission (PR: 3.1, 95%CI: 1.2–7.9; and 2.7, 1.1–7.0 respectively for promptness of gynaecological visits, and appropriateness of ultrasound examinations), low Apgar score (1.6, 1.1–1.2; 1.9, 1.3–2.7; and 2.1, 1.5–2.8 respectively for appropriateness and promptness of gynaecological visits, and appropriateness of ultrasound examinations), and low birth weight (1.8, 1.5–2.3 for appropriateness of laboratory test examinations). Benefits for mothers and newborn are expected from improving adherence to guidelines-driven recommendations regarding antenatal care even for low-risk, uncomplicated pregnancies.

## 1. Introduction

Antenatal care (ANC) aims to monitor and improve the wellbeing of the mother and foetus, detect complications, prepare for birth, and promote healthy behaviours [1,2]. Modern strategies recommend a cost-efficient approach based on routine care to all women and additional or specialised care addressed to women with high-risk, complicated pregnancies [3], being 12% of all pregnancies affecting the latter condition [4]. Guidelines have been consistently developed to provide guidance on adequate initiation of care, number of visits, and content of routine care [2,5,6,7]. However, although ANC should regard actions known to be effective in improving maternal or neonatal outcomes, there is still a lack of evidence that the content, frequency, and timing of visits in current recommendations for low-risk, uncomplicated pregnancies are effective [8].

For example, ANC standards have been recently updated by the World Health Organization [1], and now recommend eight visits, at least, for women who are considered “low risk” [9,10]. Early trials in the 1990s, however, started from a base of a higher number of antenatal visits (often up to 14) [9]. Available systematic reviews always often agreed that reduced visits did not result in detrimental clinical outcomes. However, the definition of “low risk” varies across trials/settings. This implies that recommendations, even generic ones (such as number of visits to be performed to “low risk” women—even in high-income countries) need further evidence-based reviews.

A system for assessing integrated care pathways for specific conditions, including pregnancy, is being developed by a national group of experts under the Italian Health Ministry: the monitoring and assessing diagnostic-therapeutic paths (MAP) working group [11]. Taking inspiration from World Health Organization (WHO) recommendations [1] and the British model [5], a set of indicators for measuring ANC experienced by women on low-risk pregnancy has been developed. This approach is in line with the Italian National Health Service (NHS) vision aimed of reducing inequalities and improving the health and well-being of women and children. However, as a better ANC profile did not necessarily lead to improved maternal or neonatal outcomes, a study for validating the set of indicators through the relationship with measurable outcomes was designed. In other terms, given a set of performance indicators (i.e., measurable elements of practice for which there is consensus on their usefulness for evaluating ANC quality), our study aims of assessing their ability, in a large population and in a real world setting, to identify the components associated with clinical outcomes (defined here as the validity of a performance indicator). In view of these preliminary remarks, the current paper reports methods and findings, and discusses implications, validating national indicators to compare quality of ANC of women on low-risk, uncomplicated pregnancies.

## 2. Materials and Methods

### 2.1. Data Source

All Italian citizens have equal access to health care services as part of the National Health Service (NHS). A system of Healthcare Utilization (HCU) databases allows each Italian region to manage NHS. HCU data concern a variety of information about services supplied to NHS beneficiaries (practically all citizens), including diagnosis at discharge and inpatient services provided from public or private hospitals, outpatient drug prescriptions, specialist visits, and diagnostic exams. In addition, a database reporting the Certificates of Delivery Assistance (i.e., the so-called CeDAP), providing detailed information on the mother’s socioeconomic traits, as well as medical information on pregnancy, childbirth, and child presentation at delivery, is consistently managed in all Italian regions. As a unique personal identification code is used for all databases within each region, their record linkage allows searching out the complete care pathway of beneficiaries of NHS. In order to preserve privacy, identification codes are automatically converted into anonymous codes, and the inverse process is prevented by deletion of the conversion table. Details of healthcare utilization databases in the field of mental health have been reported elsewhere [12,13,14,15,16].

Specific diagnostic and therapeutic codes used for the current study are given in Supplementary Materials (Table S1).

### 2.2. Study Cohort

Data used for the present study were retrieved from the HCU databases of Lombardy, a region of Italy that accounts for about 16% (more than ten millions) of its population. Childbirths occurred from female residents in Lombardy during the period 1 January 2015 to 31 December 2017 (being the years 2015, 2016, and 2017 denoted as the index years) were selected from the CeDAP database. Women who became beneficiaries of the regional NHS from less than two years before the last menstrual period (LMP) were excluded, to allow investigating their previous history. Exclusion was extended to women with signs suggestive of intermediate/high-risk, complicated pregnancy, the latter being qualified according to three time-related criteria: (i) the entire reproductive history (previous foetal death and stillbirth); (ii) two years before LMP (hospital admission for foetal death and stillbirth, congenital malformation, any complication of pregnancy, malignancy, radiotherapy, and dispensation of selected drugs); and (iii) current pregnancy (use of assisted reproductive technology pregnancy, multiple pregnancy, gestational age shorter than 37 weeks or longer than 42 weeks, hospital admission for miscarriage, stillbirth, congenital malformation or any complication of pregnancy, foetal growth restriction). The remaining women, who we considered at low-risk, i.e., those expected to experience an uncomplicated pregnancy, were included in the study cohort. Selected maternal traits of the study cohort including age at delivery, nationality, marital status, education, employment and parity, were considered.

### 2.3. Adherence with Recommendations

In accordance with the National System for physiological pregnancy guidelines, recommendations regarded promptness and appropriateness of number and timing of ANC, including gynaecological visits, ultrasounds, and laboratory tests [17]. Appropriateness and promptness of gynaecological visits respectively evaluated whether at least four visits were performed during pregnancy and at least one of them were carried out within the 12th week of gestation. Appropriateness of ultrasound examinations evaluated whether at least two examinations were performed during pregnancy, of whom at least one within the 12th week of gestation. Finally, laboratory test appropriateness evaluated whether controls scheduled for each pregnancy trimester were performed (details about scheduled controls are given in Supplementary Table S2). Information on gynaecological visits, ultrasounds, and laboratory tests were retrieved from the CeDAP and the outpatient service databases, where appropriate.

Other than for each individual recommendation, the cumulative number of recommendations was calculated. A score of increasing adherence was developed by categorizing each cohort woman, according to whether she complied with none or almost none (0 or 1), just some (2), almost all (3), or all (4) of the recommendations during pregnancy.

### 2.4. Outcomes

Both maternal and neonatal adverse outcomes, expected to be worse among women who did not adhere with recommendations, were considered. Maternal outcomes included those that occurred during delivery (3rd and 4th degree perineal tears) and/or in the following seven days (postpartum haemorrhage—more than 500 mL, admission in intensive care unit, hysterectomy), and/or in the following 42 days (pregnancy-related complications and hospital readmission for any cause). Women who did not have vaginal birth (i.e., those who gave birth via caesarean delivery) were excluded from the cohort for calculating the proportion of women who experienced third and fourth degree perineal tears.

Neonatal outcomes included low Apgar scores at five minutes (i.e., a score value less than 7), low birth weight (i.e., a weight less than 2500 g), and late preterm birth (i.e., between the 34th and 36th week). The expanded cohort, including women with gestational age ranging from the 34th to the 42nd week, was considered for calculating the proportion of women who experienced late preterm birth. Information on maternal and neonatal outcomes were retrieved from the CeDAP and the inpatient service database, where appropriate.

### 2.5. Statistical Analysis

Descriptive, univariate methods were used for measuring prevalence of women who were adherent with recommendations, and of maternal and neonatal outcomes.

Log-binomial regression models were fitted to estimate the prevalence ratio (PR), and corresponding 95% confidence interval (95% CI), measuring the association between exposure to no-adherence with a given recommendation and the risk of a maternal or neonatal outcome. The model that included one recommendation at a time as the only covariate, as well as the model that included other maternal features (i.e., those above –mentioned, mostly concerning maternal sociodemographic traits) were fitted, and the corresponding estimates were respectively denoted (unadjusted and adjusted ones). In addition, an overall log-binomial regression model was built by considering the association between the score of increasing adherence and the risk of each maternal or neonatal outcome while adjusting for the above-mentioned baseline data.

Several tools were used for measuring the associations of interest. First, as we aimed to test the association between four recommendations and eight outcomes (with a total, therefore, of 32 possible PR estimates), we reasoned on the high probability of generating false positive associations simply by chance (i.e., false signals). For this reason, we accounted for multiple comparisons correction by using the false discovery rate (FDR) proposed by Benjamini and Hochberg [18]. All *p*-values were reported based on 2-sided tests and *p* < 0.05 was considered statistically significant.

Second, we realized that low exposure prevalence was expected to occur in our cohort (i.e., few women did not adhere with recommendations). For this reason, a cautious approach was adopted by excluding from the calculations exposure–outcome pairs for which our sample size was not sufficient to appreciate a 2.0 minimum detectable PR, with a 0.80 power (i.e., of appreciating as significant PR values so high not being plausible).

Third, data on maternal characteristics were sometimes missing. Restricting analyses to the subset of women with all of the data observed would have resulted in a significant loss of information and possibly biased estimations. For this reason, we decided to generate appropriate values of missing data for those women with missing covariates. With this aim, the iterative procedure known as fully conditional specification (FCS) was used [19].

Fourth, as the adherence with recommendations, as well as the considered outcomes, are likely affected by relevant characters not recorded in healthcare databases such as ours, residual confounding should be taken into account. With this aim, the high-dimensional propensity score (HDPS) stratification design was adopted to account for residual confounders [19]. Exposure propensity scores were derived through the HDPS algorithm, an automated technique that identifies and prioritizes covariates that may serve as proxies for unmeasured confounders in large electronic healthcare databases [20]. Shortly, predicted probability of exposure (i.e., the propensity score of interest) was estimated for each cohort member through a logistic regression model, which included as covariates the above-mentioned baseline data, plus all of the possible causes of hospital discharge experienced, and all of the drugs prescribed to cohort members in the 2-year period prior to the index hospital admission. The 200 most predictive covariates were selected. Cohort members were then assigned into decile (i.e., 10 equal-sized) strata, according to the individual’s propensity score. A log-binomial regression model was then fitted within each stratum, and an overall estimated effect was calculated by taking the (weighted) average across strata [21].

Fifth, women who had shorter pregnancy duration (i.e., those who experienced late preterm birth) had less time to experience appropriate care (e.g., a woman who gave birth at the 34th week of gestation was less likely to receive four gynaecological visits than a woman who gave birth at the 42nd week). Since this may lead to misclassified immortal person-time [22], we did not evaluate the relations for which such a bias might be pertinent.

Finally, the robust estimator, which took into account correlations in women with multiple pregnancies during the study period, did not substantially modify the estimates; correlation structures were omitted from analyses.

## 3. Results

**Figure 1:**
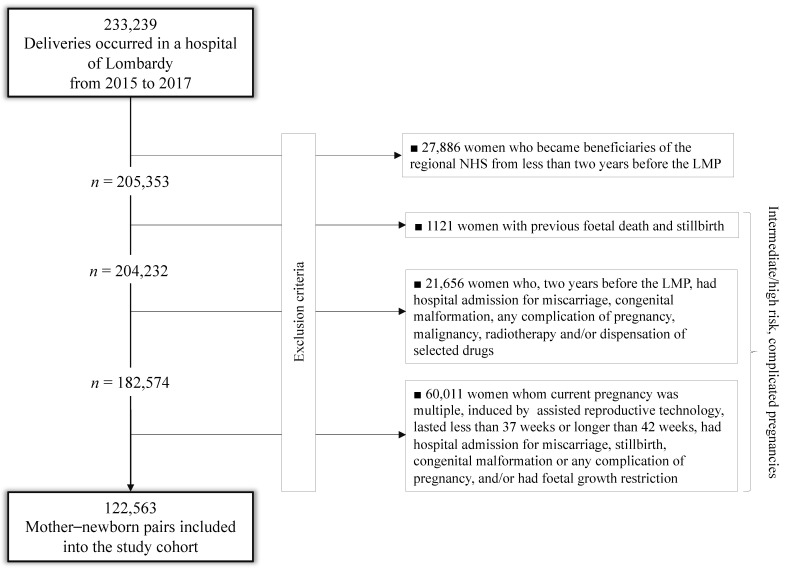
Flow-chart of inclusion and exclusion criteria.

Among the 233,239 deliveries that occurred in Lombardy from 2015 to 2017, 27,886 (12%) were excluded because the mother was an NHS beneficiary for less than two years before the last menstrual period (LMP); others 82,790 (40.3%) had signs suggestive of intermediate/high-risk, complicated pregnancies, detected from the entire reproductive history (1121), from investigations limited to two years before LMP (21,658) or from the current pregnancy (60,011). The remaining 122,563 mother–newborn pairs were included in the study cohort (Figure 1).

Table 1 shows that, with the exception of parity, there were very few missing values of the investigated traits. A small minority of women did not adhere with the considered recommendations during their pregnancy with prevalence ranging from 2.6% (laboratory tests) to 5.9% (gynaecological visits). It is noteworthy that only 176 women did not adhere to any recommendations, while just under 90% of them turned out to comply with all of the recommendations. Similarly, neonatal, and even more, maternal adverse outcomes occurred very rarely in our setting, ranging from 0.01% (postpartum hysterectomy) to 3.5% (pregnancy-related complications) (Table 2). This involved the exclusion of few exposure–outcome associations from our analysis, in particular, those investigating risk factors of hysterectomy, as well as those concerning the association between appropriateness of laboratory tests and the risk of intensive care unit transferring.

**Figure 2:**
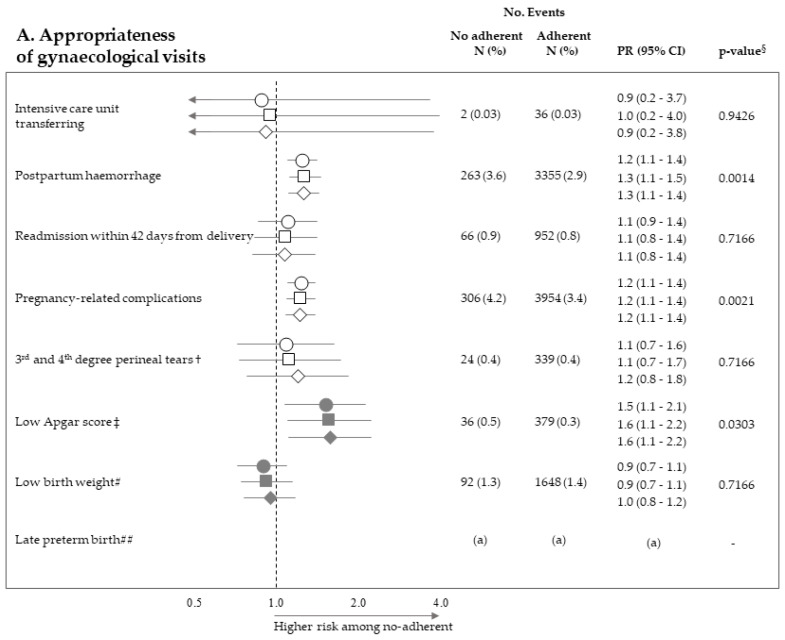

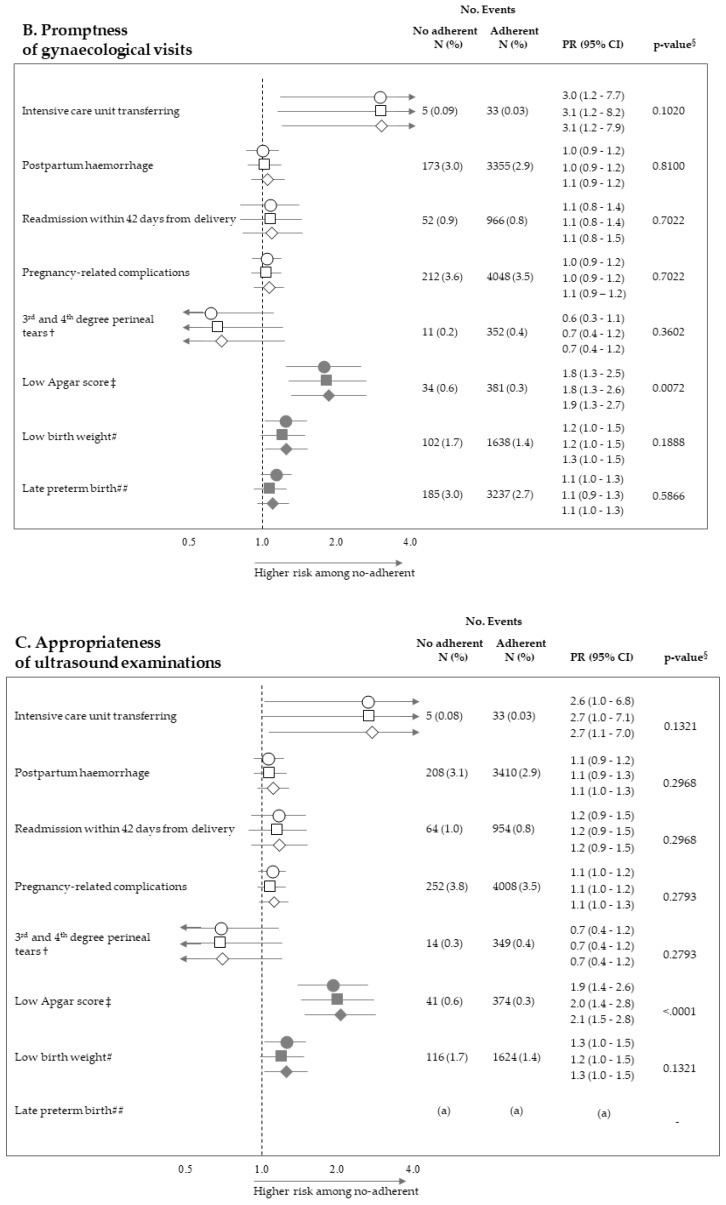

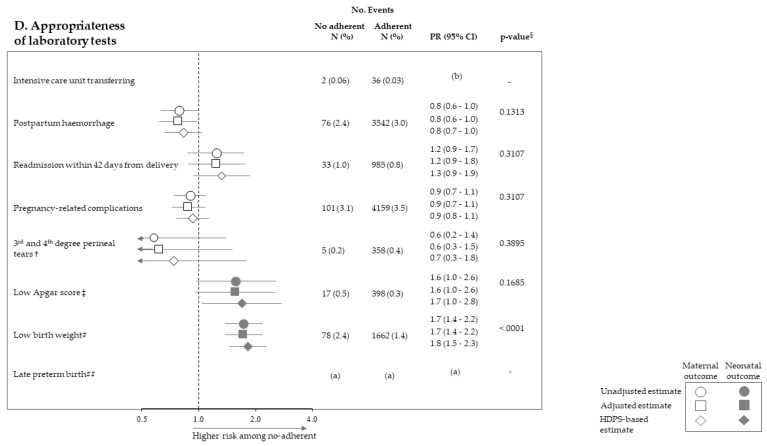
Forest plot picturing the association between exposure to no-adherence with selected recommendations and the risk of maternal or neonatal outcomes. Prevalence ratio (PR) and 95% confidence interval (CI) were estimated with log-binomial regression models. Estimates unadjusted and adjusted for covariates listed in Table 1 are shown. In addition, estimates obtained with high-dimensional propensity score (HDPS) stratification design are displayed. † The 96,837 women who experienced vaginal birth were included into the specific cohort, rather than the 122,563 ones with either vaginal birth or caesarean delivery as for other associations. ‡ Value of the Apgar score at five minutes <7. # Birth weight <2500 g. ## Late preterm birth between the 34th and 36th week. The 125,985 women with gestational age ranging from the 34th to the 42nd week were included in the specific cohort, rather than the 122,563 ones with gestational age from the 37th and the 42nd week as for other associations. § *p*-value adjusted using the Benjamini–Hochberg false discovery rate control procedure. (a) Because women who had shorter pregnancy duration had less time to experience appropriate care, and as this may lead to misclassified immortal person-time, the specific association was not evaluated (see text). (b) As exposure and outcome were both very rareness, the sample size was not sufficient to appreciate a 2.0 minimum detectable PR, and the specific association was then not evaluated (see text).

Figure 2 pictures the association between exposure to non-adherence with recommendations and the risk of maternal and neonatal outcomes. Among the 28 investigated associations, non-adherence to four recommendations resulted positive association with some outcome (i.e., they were suggestive of harmful action), while there was never evidence of negative associations (protective effects). It should be noticed that, among the investigated outcome, low Apgar score appeared as the one more sensitive to the lack of adherence with almost all recommendations, being the corresponding PR almost always significant (with the exception of appropriateness of laboratory tests) and their values higher than those of the other outcomes. On the other hand, among the considered recommendations, the lack of appropriateness of gynaecologic visits appeared to be the recommendation more predictive of the considered outcomes, being significantly associated with maternal (postpartum haemorrhage and pregnancy-related complications) and neonatal (low Apgar score) outcomes. The lack of appropriateness of laboratory tests was significantly associated with low birth weight. Adopted methods of estimates did not substantially affect the results.

Finally, a trend towards increasing prevalence of neonatal outcome (i.e., low Apgar score and low birth weight) with decreasing number of complied recommendations was observed (Table 3). Conversely, there was no evidence that the number of recommendations affected maternal outcomes.

## 4. Discussion

According to the very stringent criteria set up by our MAP group for post-hoc definition of low-risk, uncomplicated pregnancies, around 60% of the pregnancies observed in the Italian region of Lombardy, should have been candidates for “routine” ANC. The complementary figure that about 40% of pregnancies should be considered at intermediate/high-risk, a percentage enormously higher than expected 6–33% [4,23,24,25], must be considered as a very precautionary tool for advising additional specialized care addressed to pregnancy at greater risk. Our selection criteria explain that very low prevalence of adverse outcomes have been observed. In fact, observed prevalence of low (below 7) five min Apgar score, and low (below 2500 gr) birth weight, respectively of 0.3% and 1.4%, should be compared with prevalence, respectively around 0.7–1.4% [26,27] and 4–8% [28,29], reported for developed countries.

In our cohort of pregnancies classified at low-risk for complications where a low prevalence of adverse outcomes had been consequently manifested, a high proportion of women (about 88%) adhered with all of the recommendations established by guidelines. The new important finding, however, is that lack of adherence to recommendations on gynaecologic visits, ultrasounds, and laboratory tests, and was associated with several maternal and neonatal adverse outcomes. The strength of these associations was not trivial since maternal outcome prevalence observed among no-adherent women was from 20% (gynaecologic visit promptness and pregnancy-related complications) to 210% (gynaecologic visit appropriateness and intensive care unit transferring) higher than those observed among adherent women. Analogously, risk excess of adverse neonatal outcomes (in particular low-Apgar score) was observed for women who did no adhere to recommendations on appropriateness of gynaecologic visits (60%) and ultrasounds (110%). It should be emphasized that, among the 28 investigated associations, none suggested a protective action of the non-adherence to recommendations. In addition, we found that, beyond each individual recommendation, the cumulative number of complied recommendations predicted neonatal adverse outcome—that is, the higher the value, the better the protective action on Apgar score and birth weight. Among the possible explanations for this finding, the more reasonable the risk of adverse neonatal outcome might be reduced, even in low-risk, uncomplicated pregnancies by structured care, of which regular control might be a proxy. Anyhow, under the point of view of public heath, these findings taken together are very important for reaching a consensus on how to measure and compare the quality of ANC in low-risk, uncomplicated pregnancies, developing process improvements, and reducing practice heterogeneity.

One explanation of our results is that non-adherence with recommendations may be a surrogate of uncontrolled factors linked with overall health-seeking behaviour, which accompany, but are different from, a better adherence. For example, women more adherent might be more compliant with healthy lifestyle advice (e.g., correct nutrition, smoking cessation, iron, and folic acid supplementation), and might have healthier anthropometric features (e.g., acceptable body mass index during pregnancy). It should be emphasized, however, that such unmeasured factors are expected to be intermediate outcomes of better adherence. That is, women who regularly attend gynaecological/obstetric outpatient clinics, receive advice for healthier behaviour, so that these factors should mediate the action of adherence on the considered outcomes, rather than confound its effect [30,31,32,33]. Furthermore, having more visits could have led to the recognition of conditions in which more intensive monitoring of pregnancy and the foetus, and choices related to childbirth, have reduced morbidity.

Our study has strengths and limitations. It is a very large investigation reporting real-world evidence on the association between adherence with recommended ANC in low-risk, uncomplicated pregnancy, and maternal and neonatal adverse outcomes, a field which, according to our best knowledge, has never been investigated. On the other hand, random and systematic uncertainty of observational evaluations, especially in the setting of ANC, should be carefully considered. As far as random uncertainty is concerned, despite avoiding underpowered exposure–outcome associations, weak associations (say, those with a true prevalence rate lower than 2.0) likely had escaped. In addition, despite the Benjamini and Hochberg method allowed accounting for multiple testing, a power loss must to put in the bill [18]. As far as systematic uncertainty is concerned, privacy concerns prevented us from assessing the validity of the information recorded in the Certificates of Delivery Assistance, as well as the diagnostic data from hospital charts, so that misclassification of both exposure and outcome cannot be excluded. Finally, the lack of data on important factors, such as smoking, alcohol, and illicit drug use, may further contribute to some unavoidable source of uncertainty. Further evidence is thus needed to confirm the protective role of adherence to recommendations even during low-risk, uncomplicated pregnancies.

## 5. Conclusions

In our large cohort of women experiencing low-risk, uncomplicated pregnancy, the lack of adherence with recommendations established by guidelines (i.e., gynaecologic visits, ultrasounds, and laboratory tests) was associated with several maternal and neonatal adverse outcomes. This suggests that maternal and infant health might be improved even in low-risk, uncomplicated pregnancies by structured care, of which regular control, by using “administrative data” from Italian NHS databases, might be a proxy. On the policymaker point of view, tight control through regular clinical examinations must consider the cornerstones of national guidance, national audits, and quality improvement incentive schemes.

**“Monitoring and assessing care pathways (MAP)” working group (Italian Ministry of Health)**

Polytechnic University of Marche (coordinator): Andrea BUCCI, Flavia CARLE, Marianxhela DAJKO

- Italian Ministry of Health, Dept of Health Planning: Silvia ARCÀ, Donata BELLENTANI, Velia BRUNO, Simona CARBONE, Carla CECCOLINI, Angela DE FEO, Lucia LISPI, Rosanna MARINIELLO, Maurizio MASULLO, Federica MEDICI, Paola PISANTI, Modesta VISCA, Rinaldo ZANINI; Dept of health prevention: Teresa DI FIANDRA, Natalia MAGLIOCCHETTI, Giovanna ROMANO
- University of Milano-Bicocca, Laboratory of Healthcare Research & Pharmacoepidemiology: Anna CANTARUTTI, Giovanni CORRAO, Pietro PUGNI, Federico REA
- Department of Epidemiology Lazio Region: Marina DAVOLI, Mirko DI MARTINO, Adele LALLO
- Aosta Valley Region: Patrizia VITTORI, Giuliana Vuillermin
- Campania Region: Alfonso Bernardo, Anna Fusciante
- Emilia Romagna Region: Laura BELOTTI, Rossana DE PALMA, Enza DI FELICE
- Friuli Venezia Giulia Region: Roberta CHIANDETTI, Elena CLAGNAN, Stefania DEL ZOTTO, Andrea DI LENARDA, Aldo MARIOTTO, Marisa PREZZA, Loris ZANIER
- Lazio Region: Marina DAVOLI, Danilo FUSCO, Mirko DI MARTINO, Adele LALLO, Chiara MARINACCI
- Lombardy Region: Antonio LORA, Luca MERLINO
- Marche Region: Liana SPAZZAFUMO, Simone PIZZI
- Molise Region: Maria SIMIELE, Giuseppe MASSARO
- Puglia Region: Ettore ATTOLINI, Vito LEPORE, Vito PETRAROLO
- Sicily Region: Giovanni DE LUCA, Giovanna FANTACI, Sebastiano POLLINA ADDARIO, Salvatore SCONDOTTO
- Tuscany Region: Francesco BELLOMO, Mario BRAGA, Valeria DI FABRIZIO, Silvia FORNI, Paolo FRANCESCONI, Francesco PROFILI
- Veneto Region: Francesco AVOSSA, Matteo CORRADIN, Silvia VIGNA
- Research and Health Foundation (Fondazione ReS -Ricerca e Salute-): Letizia DONDI, Nello MARTINI, Antonella PEDRINI, Carlo PICCINNI
- National Agency for Regional Health Services: Mimma COSENTINO, Maria Grazia MARVULLI
- ANMCO (National Association of Hospital Cardiologists) Study Center: Aldo MAGGIONI

## Figures and Tables

**Table 1:** Sociodemographic traits, parity and adherence with selected recommendation of the 122,563 mothers included in the study cohort. Italy, region of Lombardy, 2015–2017.

Socio-Demographic Traits	No. (%)
**Age at delivery**	
16 to 25 years	12,314 (10.1)
26 to 35 years	68,365 (55.8)
36 to 45 years	41,884 (34.2)
**Birth place**	
Italian-born	90,676 (74.0)
Foreign-born	29,981 (24.5)
Missing	1906 (1.6)
**Marital status**	
Married	76,667 (62.6)
Unmarried	45,856 (37.4)
Missing	40 (0.0)
**Employment**	
Employed	87,196 (71.1)
Unemployed	35,262 (28.8)
Missing	105 (0.1)
**Education**	
Low	26,380 (21.5)
Intermediate	53,554 (43.7)
High	42,567 (34.7)
Missing	62 (0.1)
**Parity**	
Null parity	16,505 (13.5)
Multi parity	65,493 (53.4)
Missing	40,565 (33.1)
**Non-adherence to selected recommendations**	
Appropriateness of gynaecological visits	7262 (5.9)
Promptness of gynaecological visits	5868 (4.8)
Appropriateness of ultrasounds	6649 (5.4)
Appropriateness of laboratory tests	3237 (2.6)
**Number of complied recommendations**	
0	176 (0.1)
1	1251 (1.0)
2	4854 (4.0)
3	8851 (7.2)
4	107,431 (87.7)

**Table 2:** Maternal and neonatal adverse outcomes experienced from the 122,563 mother-newborn pairs included into the study cohort. Italy, region of Lombardy, 2015–2017.

	No. (%)
**Maternal adverse outcomes**	
Hysterectomy	12 (0.0%)
Transferring to the intensive care unit	38 (0.0%)
Postpartum haemorrhage	3618 (3.0%)
Readmission within 42 days from delivery	1018 (0.8%)
Pregnancy-related complications	4260 (3.5%)
3rd and 4th degree perineal tears †	363 (0.4%)
**Neonatal adverse outcomes**	
Low Apgar score at five minutes ‡	415 (0.3%)
Low birth weight #	1740 (1.4%)
Late preterm birth ##	3422 (2.7%)

† Among the 96,837 women who experienced vaginal birth. ‡ Score value less than 7. # Weight less than 2500 g. ## Gestational age between 34 and 36 weeks experienced from the 125,985 women with gestational age ranging from the 34th to the 42nd week.

**Table 3:** Trends in prevalence ratios (and 95% confidence intervals) measuring the association between decreasing number of complied recommendations and maternal and neonatal adverse outcomes experienced from the 122,563 mother–newborn pairs included into the study cohort. Italy, region of Lombardy, 2015–2017.

	Number of Complied Recommendations				*p*-Trend
	4	3	2	0 or 1	
**Maternal adverse outcomes**					
Transferring to the intensive care unit	1.0 (ref.)	1.8 (0.6 to 5.0)	3.8 (1.4 -10.1)		0.0976
Postpartum haemorrhage	1.0 (ref.)	1.2 (1.1–1.3)	1.2 (1.0–1.4)	0.7 (0.5–1.0)	0.2734
Readmission within 42 days from delivery	1.0 (ref.)	1.1 (0.8–1.4)	1.2 (0.9–1.6)	1.1 (0.6–1.9)	0.2478
Pregnancy-related complications	1.0 (ref.)	1.1 (1.0–1.3)	1.2 (1.0–1.3)	0.9 (0.7–1.2)	0.1227
**Neonatal adverse outcomes**					
Low Apgar score at five minutes †	1.0 (ref.)	1.4 (1.0–2.0)	1.6 (1.1–2.5)	3.2 (1.8–5.6)	<0.0001
Low birth weight ‡	1.0 (ref.)	1.0 (0.9–1.2)	1.2 (1.0–2.2)	1.6 (1.1–2.3)	0.0087

† Score value less than 7 ‡ Weight less than 2500 g.

## Data Availability

Restrictions apply to the availability of these data. Data was obtained from the Lombardy Region and are available with the permission of Lombardy Region.

## Supplementary Materials

The following are available online at https://www.mdpi.com/1660-4601/18/1/173/s1, Table S1: ATC, ICD-9 Diagnostic codes and outpatient codes, Table S2: Laboratory test specific outpatient codes.
