# Supplementary material for: Association between Adherence with Recommended Antenatal Care in Low-Risk, Uncomplicated Pregnancy, and Maternal and Neonatal Adverse Outcomes: Evidence from Italy
Source: Int J Environ Res Public Health. 2020 Dec 29;18(1):173. doi: 10.3390/ijerph18010173 (PMC7795028; doi:10.3390/ijerph18010173)
Supplement: Supplementary file 1 [file ijerph-18-00173-s001.pdf]

Supplementary materials

Table S1.

- ICD-9 Diagnostic codes for delivery

**ICD-9 codes**	
ICD-9 Diagnostic codes	v27.xx or 640.xy – 676.xy (where y=1 or 2)
ICD-9 Procedures codes	72.x, 73.2, 73.5, 73.6, 73.8, 73.9, 74.0, 74.1, 74.2, 74.4, 74.99

- ICD-9 Diagnostic codes for miscarriage and/or stillbirth

**ICD-9 codes**	
Miscarriage	634-639
Stillbrith	656.4, V27.1, V27.4, V27.7

- ICD-9 Diagnostic codes for congenital malformation

**ICD-9 codes**	
Congenital malformation	740 - 759

- ICD-9 Diagnostic codes for the complication of pregnancy

Diseases	ICD-9 codes
Infectious and parasitic diseases	008.8, 010-018, 042, 053.79, 054.1, 077.98, 078.88, 079.53, 079.88, 079.98, 084.0, 084.3, 090-099, V08
Neoplasms	140.0 – 239, V10
Endocrine, nutritional and metabolic diseases, and immunity disorders	240-245, 246, 250.01, 250.02, 250.82, 251.0, 277.0, 278.01
Diseases of the blood and blood-forming organs	280-284, 285 (escluso 285.1), 286, 287.5
Mental disorders	295.34, 296.41, 296.42, 296.44, 296.64, 298.3, 298.8, 298.9, 300.11, 301.82, 301.9, 307.51, 312.82
Substance abuse	303-305
Diseases of the nervous system and sense organs	323.9, 340, 345.10, 345.40, 345.90
Diseases of the circulatory system	390-398, 410-429, 431, 433, 434, 437, 438, 451.89, 453.2, 453.41, 456.8
Pneumonia and influenza	480-487
Chronic obstructive pulmonary disease and allied conditions	491-492, 494, 496
Diseases of the respiratory system	491.2, 493, 510-514, 518.81
Pneumoconioses and other lung diseases due to external agents	500-508
Other diseases of respiratory system	515-517
Diseases of the digestive system	522.6, 551.1, 552.00, 560.89, 560.9 568.81, 568.82, 573.3
Nephritis, nephrotic syndrome, and nephrosis	580-589
Diseases of the genitourinary system	614.6, 614.9, 618.2, 620.8, 620.9, 621.0, 622.12, 622.5, 624.8, 625.5, 625.8, 2626.2, 626.6, 628.0, 628.1, 628.2, 628.8, 628.9
Complications of pregnancy, childbirth, and the puerperium	632, 633.11, 634.92, 635.90, 635.91, 635.92, 640.00, 640.01, 640.03, 640.80, 640.83, 640.90, 640.93, 641, 642.01, 642.03, 642.11, 642.13, 642.21, 642.23, 642.31, 642.33, 642.41, 642.43, 642.54, 642.63, 642.64, 642.71, 642.90, 642.93, 644.00, 644.03, 644.10, 644.13, 644.20, 644.21, 645, 646.2, 646.63, 646.71, 646.73, 646.81, 646.83, 646.84, 646.91, 646.93, 647.0-647.2, 647.3, 647.43, 647.61, 647.63, 647.83, 647.93, 648.01, 648.03, 648.1, 648.23, 648.3 (escluso 648.32 e 648.34), 648.43, 648.53, 648.61, 648.63, 648.73, 648.81, 648.83, 648.93, 649.13, 649.23, 649.43, 649.44, 649.53, 651, 652, 653.53, 654.13, 654.21, 654.23, 654.43, 654.53, 654.63, 654.93, 655, 656.13, 656.33, 656.41, 656.51, 656.53,

	656.63, 656.73, 656.81, 656.83, 657.01, 657.03, 658.01, 658.03, 658.1, 658.4, 658.83, 658.93, 659.63, 659.70, 659.73, 659.83, 660.93, 661.43, 663.0, 665.93, 668.03, 668.13, 668.23, 668.94, 671.23, 671.33, 671.53, 671.83, 671.93, 674.03, V23.0, V23.2, V23.4, V23.5, V23.7, V23.8, V27.2-V27.9, V31-V37
Diseases of the musculoskeletal system and connective tissue	710
Congenital anomalies	745-747
Fetus or newborn affected by maternal complications of pregnancy	760.0, 760.1, 760.3, 761.5
Slow fetal growth and fetal malnutrition	764
Intrauterine hypoxia and birth asphyxia	768
Symptoms, signs and ill-defined conditions	780.02, 786.09, 786.50, 786.51
Injury and poisoning	852.42, 854.00, 868.00, 868.03, 995.91, 998.11
Supplementary classification of factors influencing health status and contact with health services	V10.60, V15.42, V15.5, V18.9, V21.8, V53.39, V67.59, V71.2, V71.9

- ATC, ICD-9 Diagnostic codes and outpatient codes for the prescription of drugs and radiotherapy

	**ATC codes**
Drugs used in diabetes	A10
Antithrombotic agents	B01
Cardiovascular system	C
Corticosteroids for systemic use	H02
Thyroid therapy	H03
Antineoplastic and immunomodulating agents	L
Psycholeptics and psychoanaleptics	N05 - N06

Radiotherapy	**ICD-9 Diagnostic code:** V58.0<br>**ICD-9 Procedure code:** 92.2<br>**outpatient codes:** 92.2x.x, 92.47.8, 92.47.9

Table S2.

- Laboratory tests appropriateness

**At least once during pregnancy**	Laboratory test specific outpatient codes
Blood exam complete:<br>citometric examination and leucocitary counting differential Hb, GR, GB, HCT, PLT, IND. LEAD.	90.62.2
Urine test	90.44.3

**At least once during the first trimester**	Laboratory test specific outpatient codes
Indirect Coombs test	90.49.3
Glucose	90.27.1
Rubella virus	91.26.D
Toxoplasma IgG and IGM ANTIBODY	91.09.D

**At least once during the third trimester**	Laboratory test specific code
Hepatitis B virus (HBV)	91.18.6
